# Post-herpetic Abdominal Pseudohernia With Underrecognized Characteristics of Male Predominance and Co-occurring Fatal Visceral Herniation: A Case Report and a Literature Review

**DOI:** 10.7759/cureus.87299

**Published:** 2025-07-04

**Authors:** Chinami Kishimoto, Yuhei Kitano, Takako Matsuda-Okazaki, Noritaka Oyama, Minoru Hasegawa

**Affiliations:** 1 Dermatology, University of Fukui, Yoshida, JPN

**Keywords:** hemiparesis, herpes zoster virus, intestinal herniation, motor neuron disease, subacute-onset muscle weakness

## Abstract

Post-herpetic abdominal pseudohernia (PHAP) is a rare complication associated with herpes zoster (HZ), characterized by transient paresis of the myotome ipsilateral to the affected segmental nerves. Clinically, it manifests as asymptomatic laxity and protrusion of the abdominal musculature, mimicking abdominal wall herniation. While the varicella-zoster virus (VZV) primarily affects the sensory nervous system, the incidence and prognosis of motor involvement remain poorly estimated. Despite PHAP's self-limiting nature with a favorable prognosis in most cases, the disease requires a careful differential diagnosis to exclude a variety of structural and neurological impairments that may potentially complicate clinical assessment.

In this report, we describe a 68-year-old male patient with PHAP who developed segmental abdominal paresis two weeks after completing antiviral therapy for an HZ rash on the corresponding unilateral abdominal wall. The segmental hemiparesis was managed successfully with a combined administration of oral pregabalin, a voltage-gated calcium channel inhibitor, and vitamin B12, leading to significant improvement in impaired motor function within two months. We further discuss the current understanding of PHAP with an updated literature review of 94 cases thus far reported, emphasizing underrecognized differential diagnoses and treatment necessity that may contribute to prognostic benefits.

## Introduction

Herpes zoster (HZ) is a clinical syndrome resulting from the reactivation of varicella-zoster virus (VZV), which resides latently in dorsal ganglionic neurons along the entire neuraxis. The reactivation risk may increase with aging, stress, or immunosuppression due to the collapse of cell-mediated immunity to VZV. While HZ predominantly presents with painful dermatomal eruptions, it can also bring motor complications.

Post-herpetic abdominal pseudohernia (PHAP), first described in 1896, is a rare sequela characterized by segmental motor paresis of the abdominal musculature. The disease pathology is attributed to inflammation extending to the anterior horn motor cells, adjacent motor nerve roots, or peripheral nerves. While motor involvement occurs in approximately 5% of all HZ patients, abdominal muscle paresis has been reported with a comparable or slightly lesser frequency (0.5%-5%).

Despite its self-limiting condition, PHAP often poses a diagnostic challenge, as it can clinically mimic abdominal wall herniation, neurological disorders, and metabolic myopathies. In this report, we present a case of PHAP involving the right abdominal wall corresponding to the overlying post-HZ skin lesion. We further discuss underrecognized clinical characteristics of PHAP, which may assist in improving diagnostic accuracy and complexity, distinguishing from other potential causes of abdominal wall weakness, and optimizing management strategies.

## Case presentation

**Figure 1:**
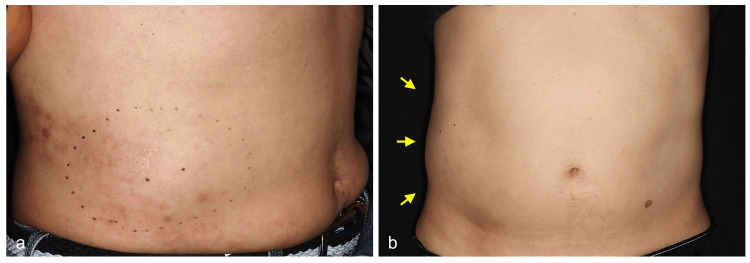
Clinical image in the standing position. Clinical appearance of the post-HZ lesion on the right abdominal wall (a), showing asymptomatic bulging corresponding to the affected area. The skin protrusion became more prominent in a standing position (b, arrows), compared to the opposite left side. HZ: herpes zoster

A 68-year-old Japanese man presented with a horizontally distributed rash on the right lateral abdomen. His medical history was significant for hypertension, hyperuricemia, and post-*Helicobacter pylori* eradication therapy. He was diagnosed with HZ by a local physician, following treatment with oral amenamevir (400 mg, once daily) and topical vidarabine ointment. Despite a gradual improvement in skin lesions (Figure 1a), he had sensory impairment in the HZ-affected dermatome just after completing antiviral therapy. Two weeks later, he suddenly developed an asymptomatic bulge in the same affected area, which became more prominent in a standing posture (Figure 1b).

**Figure 2:**
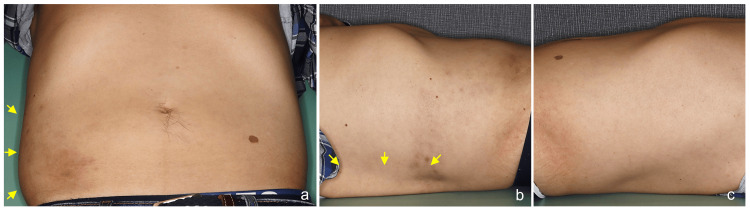
Clinical image in the supine position. Bulging of the post-HZ lesion in the supine position (a and b), which remained apparent compared to the opposite, non-affected left abdominal wall (c). Arrows indicate the protruded skin. HZ: herpes zoster

Also, a marked downward bulging of the abdominal wall was observed in the supine position (Figure 2a, 2b), particularly when compared to the opposite left abdomen (Figure 2c).

The HZ-affected skin had completely healed but remained mildly painful. Ultrasonography and computed tomography revealed no evidence of structural defects or abnormalities in the abdominal wall and intestinal tracts, as well as no abnormalities in subcutaneous and intramuscular fluid collections, masses, or bowel herniation (data not shown). Based on these clinical findings, we finally made a diagnosis of PHAP.

He was treated with oral pregabalin (150 mg, once daily), a voltage-gated calcium channel inhibitor, and mecobalamin (1000 mg, once daily), which gave significant symptomatic relief. Within two months of therapy, he obtained marked improvement in muscle tonus and complete resolution of the abdominal bulge.

## Discussion

PHAP is a rare complication of HZ associated with segmental motor paresis, specifically impairing the affected abdominal musculature. The epidemiology has been reported with increasing frequency relative to the overall incidence of herpetic motor involvement (0.5%-5%).

To ensure an updated understanding of this underrecognized clinical entity, we performed a comprehensive review of the literature using a PubMed-based search. In total, 94 individual cases with PHAP were identified [1-19]. The mean age was 65 years, with male predominance (male-to-female ratio = approximately 2.5:1). This finding differs from a previous study conducted on a region-restricted Japanese cohort, which demonstrated a slight female predominance (1:1.2) [20]. Several factors may account for this discrepancy, including a tendency of relatively abundant abdominal and visceral fat in middle-aged women. In addition, differences in the timing, frequency, and implementation strategies of HZ vaccination across different areas or countries may contribute to variations in the incidence and severity of PHAP. However, the underlying reasons for the sex differences remain inconclusive and warrant further assessment.

The most commonly affected neurosegment was Th11 (51 patients, 54%). In most cases (73%), abdominal wall paralysis occurred after the onset of HZ. The average duration of skin manifestation prior to PHAP was 26.7 days. Only seven patients (7.4%) presented with the simultaneous onset of typical HZ and pseudohernia. More interestingly, another seven patients (7.4%) presented with pseudohernia before the onset of any skin lesions, and all patients were men (Table 1) [13-19]. Pseudohernias preceding skin symptoms are uncommon but clinically important presentations, which may pose a diagnostic and management challenge in cases without typical herpes zoster lesions. Notwithstanding the unusual clinical presentation of preceding pseudohernia, their prognosis was comparable to other groups who preceded skin lesions or had both skin lesions and pseudohernia simultaneously. Among total cases, 46 patients (49%) achieved complete recovery with an average duration of 4.2 months, and the subgroup with four patients (4.3%) preceding pseudohernia exhibited a slightly shorter recovery time with an average of 3.8 months. Partial recovery was documented in 17 patients (18%), while the remaining 32 cases (34%) lacked detailed data for follow-up. Complete recovery with conservative medical management was achieved in 79.3% of the patients, with a mean period of 4.9 months in the study reviewing 36 cases.

**Table 1:** Detailed clinical summary of seven patients with PHAP, whose pseudohernia preceded the onset of any skin lesions. ND, not described; M, male; L, left; R, right; PHAP, post-herpetic abdominal pseudohernia

Age	Sex	Side	Affected segments	Constipation	Periods prior to the development of skin lesions	Outcome	Reference numbers
68	M	L	T10, T11, and T12	Pseudotumor due to paresis	1 day	Full recovery at four months	[13]
57	M​	L	T11	ND	2 weeks	Partial recovery at one month	[14]
64	M	R	T9	None	1 week	Partial recovery at two months	[15]
43	M	R	T10	ND	2 days​	ND	[16]
67	M	L	T9 and T10	ND	4 days	Full recovery at eight months	[17]
57	M	R	ND	ND	​10 days (10 years post-onset of the herpes episode)	Full recovery at one month	[18]
80	M	L​	T10 and T11	ND	3 days​	Full recovery at two months	[19]

Patients with persistent and/or intolerable symptoms may require conservative management, including supportive garments, physical therapy, and analgesia [5]. Although PHAP is generally considered a benign and self-limiting condition, serious underlying complications may coexist, particularly in the elderly. Notably, our literature review identified a co-occurrence of fatal visceral herniation in two patients, both of whom developed paralytic ileus (2.1%). Other gastrointestinal complications included severe constipation in one patient. The exact pathophysiology interconnecting PHAP to visceral herniation remains uncertain, but prolonged abdominal wall paralysis might alter intra-abdominal pressure dynamics, possibly predisposing to mechanical visceral complications. Although these complications are indeed less frequent, clinicians need to keep the secondary gastrointestinal dysfunction in mind, particularly in elderly patients or those with comorbidities.

To date, no standardized treatment and recommended guidelines for PHAP exist. Treatment strategies considerably vary, including observation, waistbands, muscle rehabilitation, neuropathic pain medications (e.g., gabapentinoids), and oral corticosteroids. Of these supportive approaches, waistbands can efficiently ameliorate the protruding abdominal skin and discomfort in the affected area. In our case, pregabalin and mecobalamin were initially prescribed once daily in accordance with the patient's preference to soften the ongoing medication burden, which included treatment for hypertension and hyperuricemia and a recent completion of *Helicobacter pylori* eradication therapy. Particularly for pregabalin, the reduced starting dose was also considered to minimize potential adverse effects such as daytime drowsiness and dizziness, which may affect the primary outpatient setting. The dosing had been planned to escalate to a standard twice-daily regimen if the initial minimum dose was tolerable and insufficient. Apart from our case, however, the effectiveness of these treatments remains inconclusive, and also, early treatment intervention with antiviral drugs does not support the preventive benefit against PHAP. Further prospective studies are needed to better define optimal management and to identify factors that may influence the prognosis of PHAP.

## Conclusions

We report an adult male case of PHAP, who achieved symptomatic relief within two months with oral gabapentinoid and vitamin B12. The disease condition is generally self-limiting with a favorable prognosis, but the presentation can mimic abdominal wall herniation, posing potential diagnostic challenges, particularly in elderly patients. Particularly in atypical or diagnostically challenging cases, clinicians should selectively consider multidisciplinary collaboration, including dermatology, orthopedics, anesthesiology, gastroenterology, and neurology. A careful differential diagnosis process is essential to exclude structural, metabolic, and neurological disorders, particularly in atypical presentations without typical skin manifestations. Conservative management, including supportive therapies and neuropathic pain medications, may facilitate early recovery. Further investigations are needed to elucidate the underlying pathophysiology, optimize treatment strategies, and establish guidelines for the early recognition and management of PHAP.
